# Genome-Wide Tissue-Specific Genes Identification for Novel Tissue-Specific Promoters Discovery in Soybean

**DOI:** 10.3390/genes14061150

**Published:** 2023-05-25

**Authors:** Lili Yu, Hao Zhang, Rongxia Guan, Yinghui Li, Yong Guo, Lijuan Qiu

**Affiliations:** The National Key Facility for Crop Gene Resources and Genetic Improvement (NFCRI)/Key Laboratory of Grain Crop Genetic Resources Evaluation and Utilization, Institute of Crop Sciences, Chinese Academy of Agricultural Sciences, Beijing 100081, China; jasmine.yu@vip.163.com (L.Y.); zhanghao03@caas.cn (H.Z.); guanrongxia@caas.cn (R.G.); liyinghui@caas.cn (Y.L.); guoyong@caas.cn (Y.G.)

**Keywords:** soybean, tissue-specific gene, transcriptome, KEGG, promoter

## Abstract

Promoters play a crucial role in controlling the spatial and temporal expression of genes at transcriptional levels in the process of higher plant growth and development. The spatial, efficient, and correct regulation of exogenous genes expression, as desired, is the key point in plant genetic engineering research. Constitutive promoters widely used in plant genetic transformation are limited because, sometimes, they may cause potential negative effects. This issue can be solved, to a certain extent, by using tissue-specific promoters. Compared with constitutive promoters, a few tissue-specific promoters have been isolated and applied. In this study, based on the transcriptome data, a total of 288 tissue-specific genes were collected, expressed in seven tissues, including the leaves, stems, flowers, pods, seeds, roots, and nodules of soybean (*Glycine max*). KEGG pathway enrichment analysis was carried out, and 52 metabolites were annotated. A total of 12 tissue-specific genes were selected via the transcription expression level and validated through real-time quantitative PCR, of which 10 genes showed tissue-specific expression. The 3-kb 5′ upstream regions of ten genes were obtained as putative promoters. Further analysis showed that all the 10 promoters contained many tissue-specific *cis*-elements. These results demonstrate that high-throughput transcriptional data can be used as effective tools, providing a guide for high-throughput novel tissue-specific promoter discovery.

## 1. Introduction

In higher plants, all cells share the same genetic information and carry out common processes, which are essential for survival during growth and development. However, they also exhibit unique functions, contributing to the morphological formation of difference tissues/organs under various environments. All these processes are strictly controlled and regulated by complex gene networks that determine the gene expression in a spatial and temporal manner as well as the expression level. These genes that express and function in particular tissues/organs or cells are called tissue-specific genes [1], and such tissue specificity is usually defined according to gene expression levels [2].

Promoters are DNA sequences located upstream of genes and contain multiple *cis*-elements that control and regulate gene expression patterns and levels [3]. Many promoters have been identified and widely applied in genetic engineering [4]. Constitutive promoters were widely used in plant genetic transformation, including cauliflower mosaic virus (CaMV) 35S promoter [5], maize ubiquitin promoter [6], and rice actin promoter [7]. These promoters express exogenous genes in almost all tissues/organs with a high activity and may cause potential negative effects by causing gene silencing, energy excessive consumption, diseases, and organ malformation [8,9]. Compared with constitutive promoters, tissue-specific promoters have remarkable advantages that can control target genes expression––in particular, development stages or tissues/organs with manipulated expression levels to avoid an excessive accumulation of heterologous proteins in untargeted tissues/organs [10,11,12]. Therefore, tissue-specific promoters are applied in improvements in agronomic traits and the production of proteins and secondary metabolites in target tissues/organs in transgenic breeding processes [13,14].

A few tissue-specific promoters have been isolated and identified in crops, including rice [8,10,15,16,17,18,19,20,21,22], maize [23,24,25], soybean [26,27,28,29,30], wheat [31,32], as well as some other species [33,34,35,36,37,38,39,40,41,42,43,44]. Among these tissue-specific promoters, the most attention has been paid to seed-specific promoters derived from the promoters of storage-protein-related genes, such as soybean [29,30], rapeseed [36], sunflower [37], and peanut [38]. Endosperm-specific, fruit-specific, and seed-coat-specific promoters have also been identified in rice and maize [45,46,47], tomato [33], and Arabidopsis [34], respectively. Other plant-tissue-specific promoters, including root- [17,27,28,38,39], pollen- [18,19,22,24,25,30,31,32,40,41], and green- tissues-specific [10,15,16,20,21,43,48,49] promoters were gradually discovered.

Green-tissue-specific promoters have great advantages in transgenic breeding [50], especially in insect and herbicide resistance breeding [51]. An increasing number of transgenic crops with insect-resistant gene expression driven by green-tissue-specific promoters have been developed. A total of five promoters expressed in nonendosperm tissues were examined in rice, and by using two of them to drive *mCry1A*, Bt protein expressed in nonendosperm tissues was barely detected in the endosperm [48]. Transgenic cotton lines carrying *PNZIP::Cry9C* showed a high expression, and Cry9C protein accumulated in green tissues, though lower in seeds [44]. The promoter of the *rbsC* gene is also a typical green-tissue-specific promoter, and by introducing the *cry2AX1* gene driven by the rbsC promoter into rice, the transgenic plant showed significant resistance to rice leaffolder with a lower level of Cry2AX1 during the tillering stage [49].

However, the number of tissue-specific promoters identified so far is limited, which cannot meet the needs of plant gene manipulation and genetic improvement. Therefore, it is necessary to explore more different types of promoters. In this study, we analyzed the expression patterns of all genes based on the transcriptome data in soybean; Kyoto Encyclopedia of Genes and Genomes (KEGG) pathway and Gene Ontology (GO) enrichment analysis were also performed. A total of 12 presumptive tissue-specific genes were further analyzed via quantitative real-time PCR (qRT-PCR), and 10 of them were identified as tissue-specific genes. Sequence analysis was carried out for the promoter regions, and the numbers of *cis*-acting elements related to tissue-specific expression were found. These results provide insight for novel native tissue-specific promoter discovery, serving plant transgenic research and crop genetic improvement.

## 2. Materials and Methods

### 2.1. Identifying Tissue-Specific Genes in Soybean

Transcriptome data of nine soybean tissues, including leaves, stems, shoot apical meristem (SAM), flowers, pods, seeds, roots, root hairs, and nodules, were downloaded from the Phytozome database (https://phytozome-next.jgi.doe.gov) (accessed on 15 March 2023) that hosts a collection of RNA-seq data of 26 experiments for soybean [52]. These data included expression information of 56,044 genes (Wm82.a2.v1), and those expressed higher in one tissue (FPKM > 100), meanwhile, unexpressed or weakly expressed (0 < FPKM < 10) in other tissues, were defined as tissue-specific genes. Heatmaps were drawn based on the normalized values of Row Z-scores by ‘Pheatmap’ in R with a clustering method of ‘complete’ and Euclidean distance.

### 2.2. Gene Ontology (GO) and Kyoto Encyclopedia of Genes and Genomes (KEGG) Enrichment Analysis

GO enrichment analysis was performed on Phytozome (https://phytozome-next.jgi.doe.gov/phytomine/begin.do) (accessed on 8 May 2023) with a reference Williams 82 genome. KEGG pathway was enriched by KOBAS 3.0 (http://kobas.cbi.pku.edu.cn/genelist/) (accessed on 10 May 2023). Both GO terms and KEGG pathways with *p*-value < 0.05 were considered as significant enrichments. Data visualization was performed using ‘ggplot2’ package in R software.

### 2.3. Plant Materials and Growth Conditions

Seeds of soybean cultivar ‘Williams 82’ were sown in a 10 × 10 × 10 cm pot with vermiculite fillers, and seedlings were grown in a growth chamber (RXZ-500D; Ningbo Jiangnan Instrument, Beijing, China) with a 16 h light and 8 h dark at 26 °C. When the trifoliate leaves of plants were fully expanded, three tissues, including leaves, stems, and roots, from ten seedlings were collected. Remaining seedlings were transferred to a glass greenhouse under natural light conditions at the Institute of Crop Sciences, Chinese Academy of Agricultural Sciences (Beijing, China). A half-strength Hoagland solution was irrigated once a week for the whole growth period. Flowers and 1–2 cm pods after flowering and dehydrated seeds after maturity from ten plants were collected. In total, six tissues were stored at −80 °C for total RNA extraction.

### 2.4. Total RNA Extraction, cDNA Synthesis, and Quantitative Real-Time PCR (qRT-PCR)

Total RNA was extracted from each sample using TRIzol Reagent (Invitrogen, Carlsbad, CA, USA) following the instruction. RNase-free DNase I (New England Biolabs, Beverly, MA, USA) was used for removing genomic DNA. The cDNA was synthesized with the PrimeScript RT reagent Kit (Perfect Real Time) (Takara, Shiga, Japan). Twenty-times dilution of each cDNA product was prepared for the next step. Gene-specific primers (Table S1) were designed with Primer Premier 5.0 software (Primer-E Ltd., Plymouth, UK). qRT-PCR was performed using TB Green Premix Ex Taq (Tli RNaseH Plus) (Takara, Shiga, Japan) in an Applied Biosystems 7500 Real-Time PCR system (Applied Biosystems, Foster City, CA, USA). The gene expression level was normalized using the housekeeping gene *GmActin*. and calculated via 2^−∆CT^ method. Student’s *t*-test was used to determine the statistically significant differences.

### 2.5. Cis-Regulatory Element Analysis of Promoter Sequences

The 3 kb upstream sequence from the initiation codon of each tissue-specific gene (Wm82.a2.v1) was obtained from Phytozome database (https://phytozome-next.jgi.doe.gov) (accessed on 16 April 2023). The Plant Cis-Acting Regulatory DNA Elements (New PLACE) database (https://www.dna.affrc.go.jp/PLACE/?action=newplace) (accessed on 20 April 2023) was used for *cis*-regulatory element identification of each promoter sequence.

## 3. Results

### 3.1. Tissue-Specific Gene Identification from Soybean

**Figure 1:**
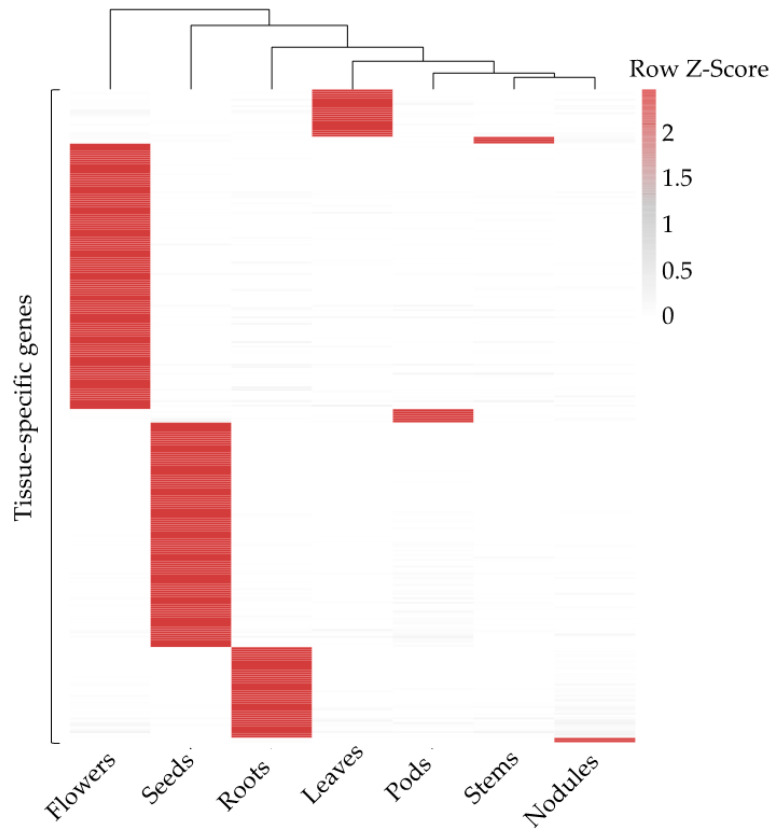
Heatmaps of 288 tissue-specific gene expression levels in seven soybean tissues, including leaves, stems, flowers, roots, nodules, pods, and seeds. The FPMK value from transcriptome data provided by Phytozome database were normalized according to Row Z-score and displayed using ‘Pheatmap’ package in R with a clustering method of ‘complete’ and Euclidean distance.

**Figure 2:**
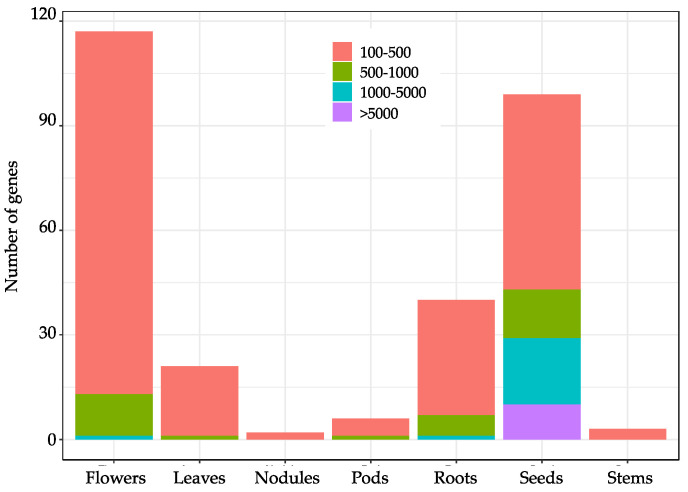
Number of different tissue-specific genes with different transcription levels (FPKM value) identified based on the transcriptome data in seven tissues, including leaves, stems, flowers, roots, nodules, pods, and seeds. Color indicates the degree of expression level, with FPKM value of 100–500 (red), 500–1000 (green), 1000–5000 (blue), and >5000 (purple).

The soybean transcriptome profiles obtained from Phytozome database were used for the identification of tissue-specific genes, including 56,044 transcripts. A total of nine tissues, leaves, stems, shoot apical meristem (SAM), flowers, pods, seeds, roots, root hairs and nodules were investigated in soybean cultivar ‘Williams 82’. Among all transcripts, 3731 genes (6.66%, 3731 out of 56,044) were found to have minor expression in all tissues. The expression levels of the 52,313 genes with detected transcripts were further analyzed for tissue-specific genes identification. Totally, 288 genes showed a tissue-specific expression pattern, and the largest number was 117 in flowers, followed by 99 in seeds, 40 in roots, and 21 in leaves. Two nodule-specific and three stem-specific genes were examined, respectively. While no SAM-specific or root-hair-specific gene was found (Figure 1 and Table S2), only 3.47% seed-specific genes showed extremely high expression with FPKM values above 5000. There were 19 seed-specific, 1 root-specific, and 1 flower-specific gene(s) (7.29% of genes) expressed at a higher level (1000 < FPKM < 5000). More than 84% of tissue-specific genes in seven tissues were expressed at a level, with an FPKM value ranging from 100 to 500 (Figure 2 and Table S2). These results indicated that some genes might be expressed with an extreme pattern in seeds.

### 3.2. GO and KEGG Enrichment Analysis of Tissue-Specific Genes

**Figure 3:**
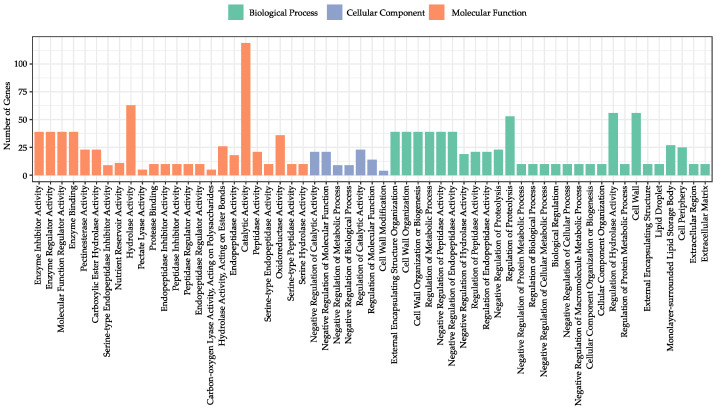
Distribution of the corresponding gene ontology (GO) terms of 288 tissue-specific genes. The enrichment of significant GO terms (*p*-value < 0.05) and their descriptions was obtained from Phytozome with a reference Williams 82 genome. Bar charts showing significant GO terms for biological process (green), cellular component (blue), and molecular function (orange). Bars were ordered by their *p*-values within each of the three groups.

Gene ontology (GO) Enrichment of 288 tissue-specific genes were performed at Phytozome database, and 59 GO terms were significantly enriched (*p*-value < 0.05). Among these GO terms, 45.16% (28/59) were enriched in the biological process, and the most significant enrichments were found in the negative regulation of catalytic activity (GO:0043086) and the negative regulation of molecular function (GO:0044092). In total, seven GO terms were characterized in the cellular component, involved in the cell wall (GO:0005618), external encapsulating structure (GO:0030312), lipid droplet (GO:0005811), monolayer-surrounded lipid storage body (GO:0012511), cell periphery (GO:0071944), extracellular region (GO:0005576), and extracellular matrix (GO:0031012). When focusing on molecular function, 24 GO terms were found, with the top three significant GO terms being enzyme inhibitor activity (GO:0004857), enzyme regulator activity (GO:0030234), and molecular function regulator activity (GO:0098772) (Figure 3 and Table S3).

**Figure 4:**
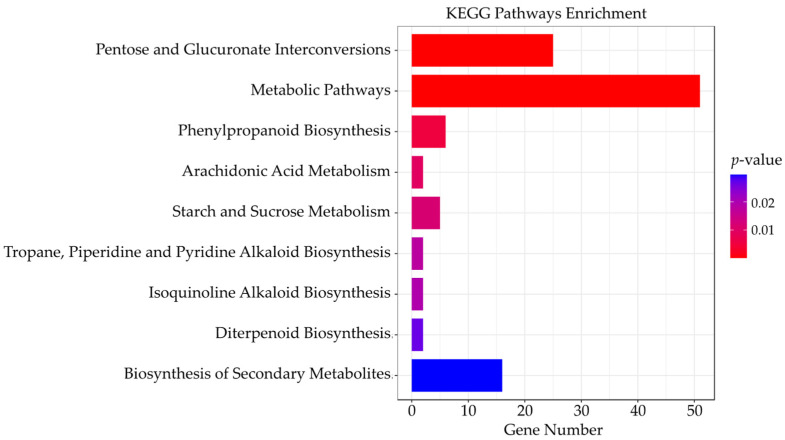
Significant enriched KEGG pathways for candidate tissue-specific genes.

Among the 288 genes, a total of 52 genes, including 29, 12, 7, 3 and 1 of flower-, root, seed-, leaf-, and pod-specific genes, had been significant enriched in nine KEGG pathways according to the KEGG pathway database (Figure 4). The highest enrichment appeared at the gmx00040 (pentose and glucuronate interconversions) of 25 genes in the flower and root. The pentose and glucuronate interconversions pathway had been reported as being involved in male sterility in cotton [53], stress [54], and pathogen infection adaptations [55] in Arabidopsis and woody plants, respectively.

One pathway, gmx00500 (the starch and sucrose metabolism pathway), was found in vegetative tissues. Starch is synthesized in the chloroplast [56], whereas sucrose is formed in the cytoplasm [57]. Photosynthetic starch/sucrose formation occurring in leaves was related to photosynthetic and nitrogen source [58]. The gmx00904 (diterpenoid biosynthesis) pathway was enriched in two seed-specific genes, which were involved in regulating rice seed storability [59]. When focusing on the root, a specific pathway, gmx00590 (arachidonic acid metabolism) was significantly enriched. Previous reports showed that arachidonic acid acted as a signaling molecule in plant stress signaling networks and impacted the rhizosphere microbial community [60,61].

### 3.3. Relative Expression Patterns of 12 Tissue-Specific Genes via qRT-PCR

**Figure 5:**
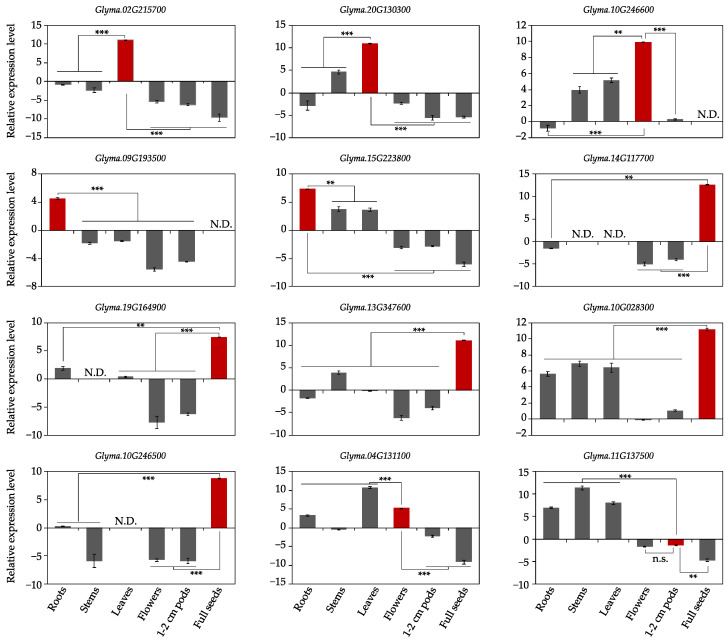
Expression analysis of 12 tissue-specific genes in different tissues of soybean via qRT-PCR. The trifoliate leaves (leaf), stem, and root when the first trifoliate leaves of plants were fully expanded, flowers and 1–2 cm pods after flowering, and dehydrated seed (full seed) after maturity from ten plants were collected. Y-axis represented log2-transformed relative expression level with a reference gene *GmACTIN*. Significance between target (red color) and other tissues (grey color) was determined by Student’s *t*-test. *** *p*-value < 0.001; ** *p*-value < 0.01; n.s. means non-significant. N.D., Not Detected.

A total of 12 genes, with a higher expression in target tissue but less accumulation in other tissues, were selected for expression pattern analysis through qRT-PCR (Figure 5). The expression patterns of ten genes were consistent with the transcriptome data. Two leaf-specific genes, Glyma.02G215700 and Glyma.20G130300, were both expressed specifically in leaves. Glyma.02G215700 showed a significantly higher expression level in leaves than other tissues, while trace mRNA was detected in stems for Glyma.20G130300. The expression of Glyma.10G246600, one of the two flower-specific genes, was significantly higher in flowers than in leaves, stems, and other tissues. Despite root-specific expression, Glyma.09G193500 and Glyma.15G223800 showed a lower expression level compared to other tissue-specific genes. All of the five seed-specific genes were significantly expressed in seeds, with varying expression levels. The most abundantly expressed gene was Glyma.14G117700, 36.7-fold higher than Glyma.19G164900, the lowest expressing gene among five seed-specific genes. Another three genes, Glyma.13G347600, Glyma.10G028300, and Glyma.10G246500, were expressed 12.2-, 13.4-, and 2.6-fold higher than Glyma.19G164900. The flower-specific gene Glyma.04G131100 and pod-specific gene Glyma.11G137500 were not target-tissue-specific. Glyma.04G131100 had a leaf-specific expression pattern, and Glyma.11G137500 was more highly expressed in stem than other tissues (Figure 5).

### 3.4. Identification of Tissue-Specific cis-Elements in Ten Promoters

**Figure 6:**
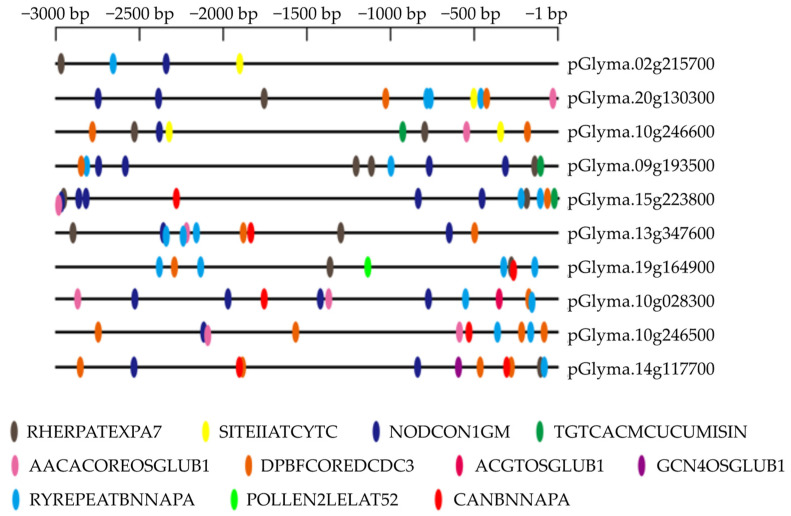
Distribution of *cis*-elements related to tissue-specific expression in ten tissue-specific promoter regions. Tissue-specific elements: RHERPATEXPA7 (KCACGW), root-hair-specific *cis*-elements [62]; SITEIIATCYTC (TGGGCY), anther- and meristem-specific expression [63]; NODCON1GM (AAAGAT), nodule-specificity [64,65]; TGTCACACMCUCUMISIN (TGTCACA), fruit-specific expression [66]; AACACOREOSGLUB1 (AACAAAC), endosperm-specific enhancement [67]; DPBFCOREDCDC3 (ACACNNG), embryo-specification elements [68]; ACGTOSGLUB1 (GTACGTG) and GCN4OSGLUB1 (TGAGTCA), endosperm-specific expression [67,69]; RYREPEATBNNAPA (CATGCA), seed-specific expression [70]; POLLEN2LELAT52 (TCCACCATA), pollen-specific activation [71]; and CANBNNAPA (CNAACAC), embryo- and endosperm-specific transcription [72].

For *cis*-element analysis, the sequences of ten tissue-specific promoters (3 kb genome sequence upstream from the ATG) were retrieved in the New PLACE database. A total of 11 kinds of tissue-specific *cis*-elements were found (Figure 6). Several other tissue-specific elements existed in two leave-specific promoters pGlyma.02g215700 (with root-hair-, seed-, nodule-, and anther- and meristem-specific elements) and pGlyma.20g130300 (with four kinds of element in pGlyma.02g215700 as well as embryo- and endosperm-specific elements). Compared with pGlyma.20g130300, fruit-specific element was identified instead of the seed-specific element. Nodule- and root-hair-specific *cis*-elements harbored in both root-specific promoters. All of the five seed-specific promoters, pGlyma.10G028300, pGlyma.13G347600, pGlyma.14G117700, pGlyma.10G246500, and pGlyma.19G164900, contained seed-, embryo-, and endosperm-specific elements with a high proportion (71.2%) of all the elements. The pollen-specific element was detected in seed-specific promoter pGlyma.19G164900; anther- and meristem-specific elements existed in not only flower- but also two leaf-specific promoters. Embryo- and endosperm-specific elements occurred in nine promoters, except pGlyma.02g215700; and a nodule-specific element was found in nine promoters, except pGlyma.19G164900.

## 4. Discussion

Previous reports showed that transcriptome profiling has been successfully applied to the genome-wide discovery of tissue-specific genes or promoters in maize [73], rice [74], and tomato [75]. The same strategy was applied in this study to identify tissue-specific genes in soybean based on the gene expression data from the Phytozome database that hosted a collection of RNA-seq expression studies acquired from several internal and external sources [76,77,78,79]. Meanwhile, we identified 27 seed-specific genes expressed in a series of developmental stages from Soybase (https://www.soybase.org (accessed on 10 May 2023)), another database for soybean genetics and genomics research, and only 7 of the genes were identified in both databases. This inconsistency was possibly due to the samples and algorithms differences occurring in the data consolidation process for Phytozome. Moreover, 24% (69/288) of these tissue-specific genes due to a higher expression activity were also identified by analyzing 1298 RNA-seq samples with different expression activity gradients [80], suggesting the possibility of a promoter with higher transcription regulatory activity.

More tissue-specific genes were detected in flower and seed rather than root, stem, leaves, pod, and nodules. This may be related to the biological function of each tissue/organ. New organs were generated from a population of stem cells at the shoot and root apex that was maintained post-embryonically [81]. Flowers and seeds appeared during the reproductive phase with unique functions distinguished from other organs. Double fertilization was accomplished in flower, and the nutrient substances were transported to soybean seeds finally, which were not replaceable. The network of uptake and transport of inorganics and organics were carried out through vascular tissue [82], and it usually existed in all vegetative tissues in shoots and roots. Compared with other tissues, studies about seed-specific expression in soybean have been widely reported [14,83,84,85,86,87,88].

The expression patterns and levels performed using transcriptome and qRT-PCR were not exactly the same, with an 83.33% (10/12) consistency. This result may be due to the genetic heterogeneity in Williams 82 [89]. Genetic variation presented within cultivars or varieties, and similar results have also been reported in tomato that 60.00% (15/25) fruit-tissue-specific unigenes obtained from transcriptome datasets were verified through qRT-PCR [75].

We focused on the unique molecular function of each kind of tissue-specific gene. For seed-specific genes, seven significant enriched GO term, including GO:0004867 (serine-type endopeptidase inhibitor activity), GO:0045735 (nutrient reservoir activity), GO:0002020 (protease binding), GO:0004866 (endopeptidase inhibitor activity), GO:0030414 (peptidase inhibitor activity), GO:0061134 (peptidase regulator activity), and GO:0061135 (endopeptidase regulator activity) were screened out, which all play roles in seed aging [90], storage proteins [91], seed germination under salt stress [92], and different nitrogen levels [93]. GO:0030570 (pectate lyase activity) and GO:0016837 (carbon-oxygen lyase activity, acting on polysaccharides) had been screened out in the flower-specific genes in the process of pigmentation in the petals [94], petal abscission [95], female cone development, and pollination mechanism [96]. The GO enrichment analysis demonstrated that the predicted tissue-specific genes have unique functions consistent with the tissue they were expressed in, with similar results also declared in a previous study [73].

The distribution of tissue-specific *cis*-elements did not exactly coincide with the promoter property. ACGTOSGLUB1 and GCN4OSGLUB1 (endosperm-specific element) were widely studied in rice and wheat [67,69,97,98]. Soybean, as a dicotyledonous plant, was distinguished from monocotyledon in the regulation of plant growth and development. Another seed-specific element, RYREPEATBNNAPA (CATGCA) [44,70], may contribute to seed-specific expression in soybean. However, it existed in other kinds of tissue-specific promoters, indicating that there were undiscovered seed-specific *cis*-elements. Genetic diversity across the plant genome were reflected in both coding sequence and noncoding region (intron, promoter region, and untranslated regions). Sequence polymorphism in the promoter, especially in the key *cis*-element, may influence the gene expression level through strong or weak binding of transcription factors [99]. Studies have shown that the promoter region contributed to the agronomic traits of plants [100,101,102,103]. By using CRISPR-Cas9, promoter editing was successfully applied to crop genetic improvements [94,95,96,97,98,99,100,101,102,103,104,105,106,107]. Together with being controlled at the transcriptional level, gene expression was also regulated at the post-transcriptional level, such as methylation [108]. Combined analysis of transcriptome and DNA methylome may provide new insights for tissue-specific expression pattern studies.

## 5. Conclusions

Promoters are widely used in plant biology and biotechnology as important molecular tools. By driving the expression of genes in plants, host plants can gain corresponding traits that are beneficial to agricultural breeding or transgenic technology research. However, currently available promoter resources are still very limited, and more types of promoters are needed for genetic improvement and application in breeding or gene function analysis. The high-throughput dataset obtained from transcriptome analysis, combined with genomics and proteomics, provides a very feasible strategy for the mining of new tissue-specific promoters. In this study, 288 tissue-specific genes expressed in different tissues were found to be enriched in nine KEGG pathways. Among them, a total of ten genes were highly expressed in target tissues, with few accumulations in other tissues identified via qRT-PCR analysis. Many *cis*-acting elements associated with tissue-specific expression were found in ten tissue-specific promoters. These results lay a foundation for further study of promoter regulation models, gene function, and transgenic breeding.

## Data Availability

All data is included in this paper and Supplementary Materials.

## Supplementary Materials

The following supporting information can be downloaded at: https://www.mdpi.com/article/10.3390/genes14061150/s1, Table S1. Primers used for qRT-PCR analysis; Table S2. The expression levels of 288 different tissue-specific genes; Table S3. The gene ontology (GO) enrichment analysis of 288 different tissue-specific genes.
