# Supplementary material for: Genome-Wide Tissue-Specific Genes Identification for Novel Tissue-Specific Promoters Discovery in Soybean
Source: Genes (Basel). 2023 May 25;14(6):1150. doi: 10.3390/genes14061150 (PMC10298445; doi:10.3390/genes14061150)
Supplement: Supplementary file 1 [file genes-14-01150-s001.zip › Table S1. Primers used for qRT-PCR analysis.pdf]

Supplementary file S1. Primers used for qRT-PCR analysis.

Primer ID	Primer sequence (5' to 3')	Gene ID
RT_Fl2466F	GATGAATGCTTCGGTTATTGCTG	*Glyma.10G246600*
RT_Fl2466R	GGCGGTGTTCGTAGTCTTCCT	
RT_Lf1303F	GGAAACTAAGAGGGTTGTGGAG	*Glyma.20G130300*
RT_Lf1303R	GTCACCACCCGTTTCACTTAT	
RT_Lf2157F	CACTTTGACGCCGACGAA	*Glyma.02G215700*
RT_Lf2157R	GCACTGCCACAGATTCAAGGT	
RT_Rt1935F	CGAGTTCTTAGGCAACATTTCA	*Glyma.09G193500*
RT_Rt1935R	CTCCCTGCTTCCATTTCCT	
RT_Rt2238F	ACTCTCTTTATGCTCATTCCAACC	*Glyma.15G223800*
RT_Rt2238R	TTCCCTGTGCTGTTATGGTGTT	
RT_Sd0283F	CCTGTCTCTGTCTCTACTCCTGG	*Glyma.10G028300*
RT_Sd0283R	AATACTTCCCTCGTTCTGTTGAT	
RT_Sd1177F	CACAAAGTCAAACCCTCCTCAAT	*Glyma.14G117700*
RT_Sd1177R	CAAGGTTCATAGCAGAAATCCGT	
RT_Sd1649F	CGTCGTGGACAGGCAGATAGT	*Glyma.19G164900*
RT_Sd1649R	CTCTTCCGTGGGTGGGCT	
RT_Sd2465F	CTAAAAGAACAGCAACAGGGAGA	*Glyma.10G246500*
RT_Sd2465R	CGTTGACCACAAATGGATAAGC	
RT_Sd3476F	CTGCCGTCAGTCAAGTCGTC	*Glyma.13G347600*
RT_Sd3476R	GGGTGAAGCACGCTAAGATG	
RT_Fl1311F	GTCTACACTCGCCCTCTTGATGC	*Glyma.04G131100*
RT_Fl1311R	GTTGCTCCATCACTTGAGGGTAT	
RT_Pd1375F	CATCTTTGGTGAGTGGTGGAAC	*Glyma.11G137500*
RT_Pd1375R	GAAAGTGTCCGTGTCTGTCTCGT	
RT_ActinF	ATCTTGACTGAGCGTGGTTATTCC	*GmACTIN*
RT_ActinR	GCTGGTCCTGGCTGTCTCC	
